# Combination of anlotinib and second-line chemotherapy as surrogate to reduce immunosuppression in patients with advanced non-small cell lung cancer

**DOI:** 10.12669/pjms.40.7.9681

**Published:** 2024-08

**Authors:** Zhi Lou, Xin Wang, Chenxi Hu, Weixuan Liu, Yajun Ji

**Affiliations:** 1 Zhi Lou, Department of Oncology, Lianyungang First People’s Hospital, Lianyungang, Jiangsu Province 222000, P.R. China; 2 Xinxi Wang, Department of Oncology, Lianyungang First People’s Hospital, Lianyungang, Jiangsu Province 222000, P.R. China; 3 Chenxi Hu, Department of Oncology precision laboratory, Lianyungang First People’s Hospital, Lianyungang, Jiangsu Province 222000, P.R. China; 4 Weixuan Liu, Department of Oncology, Lianyungang First People’s Hospital, Lianyungang, Jiangsu Province 222000, P.R. China; 5 Yajun Ji, Department of Oncology, Lianyungang First People’s Hospital, Lianyungang, Jiangsu Province 222000, P.R. China

**Keywords:** Anlotinib, Non-small cell lung cancer, Immunosuppression, Immune cells, Inflammatory cytokines, Second-line chemotherapy

## Abstract

**Objective:**

To study the clinical effects of anlotinib combined with second-line chemotherapy (SLC) on immunosuppression in patients with advanced non-small cell lung cancer (NSCLC).

**Methods:**

In this retrospective study, the medical records of 106 patients with advanced NSCLC admitted to the Lianyungang First People’s Hospital from November 2020 to March 2022 were retrospectively analyzed. Amongst 106 patients, 53 patients received second-line single-agent chemotherapy regimens (SLC group), and 53 patients received anlotinib combined with SLC (ASLC group). Prognosis, levels of immune cells and inflammatory cytokine, and adverse reactions were analyzed.

**Results:**

Clinical efficacy of the ASLC group was significantly higher than the SLC group (p<0.05). After treatment, patients in the ASLC group exhibited significantly higher levels of CD4+/CD8+ and CD4+ compared to those in the SLC group (p<0.05), while the difference in CD8+ level between the two groups was not statistically significant (p>0.05). After treatment, levels of tumor necrosis factor-α (TNF-α), interleukin-10 (IL-10), interleukin-8 (IL-8), interleukin-6 (IL-6) in the ASLC group were lower compared to the SLC group (p<0.05).

**Conclusion:**

In patients with advanced NSCLC, anlotinib combined with SLC is associated with higher levels of immune cells and reduced inflammatory factors. This treatment regimen, thus, can reduce immunosuppression and improve the prognosis of NSCLC patients.

## INTRODUCTION

Non-small cell lung cancer (NSCLC) accounts for about 80.0% of all lung cancer cases.^1^ Due to the complicated pathogenesis of NSCLC and the lack of early symptoms, most NSCLC patients are diagnosed with stage IIB–IV, past the optimal timing of surgical resection.^2^,^3^ Due to the complex pathogenesis of non-small cell lung cancer, high intra-tumor heterogeneity, atypical early symptoms and lack of effective early screening methods, most patients with lung cancer are in local advanced stage or have distant metastases and cannot undergo radical surgical resection.

Platinum-based chemotherapy (PBC), which can decelerate tumor growth and impede disease progression, is currently the preferred treatment for NSCLC.^4^,^5^ However, studies have shown that while PBC can extend the survival duration and enhance the life quality of NSCLC patients, it does not have a curative effect.^6^,^7^ Some patients also develop post-chemotherapy drug tolerance that leads to cancer recurrence.^7^ At present, no agreed standard approach exists for retreatment after failure of first-line chemotherapy (FLC), and patient survival after second-line therapy (SLT) remains extremely low.^8^ Therefore, it is crucial to explore treatment options for patients with advanced NSCLC who have failed first-line treatment.

Anlotinib is a new oral multi-target tyrosine kinase inhibitor (TKI) that can effectively inhibit vascular endothelial growth factor receptors (VEGFR), and is used for the treatment of advanced NSCLC.^9^ Anti-angiogenic drugs can normalize blood vessels, improve the tumor microenvironment, and promote immune cells and lymphocytes to enter tumor tissues more easily. Moreover, VEGFR-TKIs combined with chemotherapy can promote the transport of chemotherapy drugs into tumor tissues, thus improving the effect of chemotherapy. VEGFR-TKIs combined with chemotherapy have been widely used clinically in the treatment of patients with advanced NSCLC. However, there are few studies on anlotinib combined with chemotherapy as a second-line treatment for patients with NSCLC. Therefore, this study aimed to explore the clinical effects of anlotinib combined with second-line chemotherapy (SLC) on the levels of inflammatory factors and immune cells in patients with advanced NSCLC.

## METHODS

In this retrospective study, the medical records of 106 patients (59 males and 47 females) with advanced NSCLC, who were treated in Lianyungang First People’s Hospital from November 2020 to March 2022 were analyzed.

### Ethical Approval

The ethical approval was taken from the Ethics Committee of Lianyungang First People’s Hospital (No. LW-20240123001-01). Patient consent was waived due to the retrospective nature of the study.

Average age of the patients was (59.23±6.42) years old. Patients were divided into two groups: SLC group (n=53) and ASLC group (n=53). The SLC group received standard SLC, and the ASLC group received anlotinib combined with SLC. The diagnostic criteria of NSCLC were based on the lung mass present on pulmonary computer tomography (CT) scan and confirmed by cytology or pathological analysis.

### Inclusion criteria:

- Age 18-75 years old.
- NSCLC confirmed by pathology or cytology.^10^
- Patients with stage IIB IIIb-IV.
- Foci detectable via imaging.
- Acquired resistance to drug or recurrence subsequent to FLC.
- Disease progression after first-line TKI treatment, presence of anaplastic large-cell lymphoma kinase, ROS1 mutations, or epidermal growth factor receptor, no previous history of systemic chemotherapy, negative T790M by secondary gene test.
- Eastern Cooperative Oncology Group (ECOG) score of 0-2 points.
- No allergic reactions to the medications used in the study.
- Estimated survival time > six months.

### Exclusion criteria:

- Patients with poor blood pressure control.
- Patients who have had a thrombotic event within the past six months.
- Complicated with other malignant tumors or had distant metastases.
- History of anti-vascular targeted drug therapy.
- Patients complicated with autoimmune system diseases.
- Insufficiency or severe dysfunction of kidney, liver, heart and other important organs.
- Intolerance for allergic constitution or chemotherapy.
- Impaired cognition or abnormal behavior.

### ASLC group (anlotinib combined with chemotherapy regimen)

Patients were given anlotinib capsules (Chiatai Tianqing Pharmaceutical Group, National Approval No. H20180004, specification: 12mg/granule) orally prior breakfast. The starting dosage was 12mg administered once daily, and was discontinued a week after continuous application of two weeks. In case of intolerance to the 12 mg dosage, the dosage was adjusted to 8 mg or 10 mg daily, and treatment continued until the patient reached complete intolerance or disease progression. Throughout this period, patients received monotherapy (pemetrexed 500 mg/m^2^ in 1^st^ day or docetaxel 75 mg/m^2^ in 1^st^ day). The treatment cycle lasted for 21 days.

### SLC group (standard second-line chemotherapy (SLC)

Patients were intravenously infused pemetrexed 500 mg/m^2^ (Jiangsu Hengrui Pharmaceuticals Co, Ltd., National Approval No.H20133216, specification: 0.2g) within 0 minute to 40 minutes. The medication was administered once every three weeks, constituting one cycle, and four treatment cycles were administered.

During the treatment, patients’ conditions and adverse reactions were closely monitored. Symptomatic treatment was provided for patients experiencing Grade 1-2 adverse reactions. In case of Grade 3-4 adverse drug reactions, the drug dosage was decreased or stopped. The duration of the treatment in both groups was 12 weeks.

### Observation indicators

Venous blood after six months of therapy were analyzed. A volume of 5mL of venous blood was collected from the patients while fasting, and the serum sample was stored at - 80°C for subsequent analysis of following:

### (1) Levels of immune cells

CD4^+^, CD8^+^ and CD4^+^/CD8^+^ were assessed using flow cytometry. Following the placement of serum samples at room temperature, an equivalent volume of 2% paraformaldehyde was added and incubated for 15 minutes. Platelets were then suspended in PBS-EDTA buffer, washed twice, and 100 μl of platelet suspension was analyzed by flow cytometry. The levels of immune cells were evaluated using Cellquest software.

### (2) Levels of inflammatory factor

Serum levels of TNF-α, IL-10, IL-8, and IL-6 were assessed using enzyme-linked immunosorbent assay (ELISA), with the kits from Wuhan Boster Biological Technology, LTD. The absorbance was measured using ELISA reader (Agilent, USA) at the wavelength of 450 nm.

The adverse events were assessed based on the Common Terminology Criteria for Adverse Events v4.0 (CT-CAE 4.0), including leukopenia, nausea and vomiting, alopecia, and liver function damage. The adverse events were categorized into grades 0–IV, with higher grades indicating more serious adverse reactions.^11^

The clinical effect was assessed after four cycles of chemotherapy, and was categorized into four levels based on the Response Evaluation Criteria in Solid Tumors version 1.1 (RECIST 1.1).^12^

### Complete remission (CR)

complete disappearance of tumor, and the remission lasts ≥ one month;

### Partial relief (PR)

the product of the tumor’s maximum diameter and maximum vertical diameter decreased by over 50%, and the remission lasted ≥ four weeks;

### Stable disease (SD)

The total length of the lesions increased without reaching PD or decreased without reaching PR;

### Progression of disease (PD)

the product of tumor’s maximum diameter and maximum vertical diameter, increased by over 25%, or appearance of new lesion.

Finally, response rate was calculated as below:

Response rate = cases of (CR+PR)/total cases ×100%

### Statistical Analysis

SPSS 23.0 was used for analysis. Normally distributed measurement data were presented as mean ± standard deviation (*χ̅*±*S*), and were compared using *t* test. Counting data were presented as number and percentage (n, %), and were compared using Chi-square test. A p-value ≤0.05 was considered statistically significant.

## RESULTS

A total of 106 (N) patients were included in this study. There were 53 cases in the SLC group and 53 cases in the ASLC group, with no statistically significant difference in general data between the groups (p>0.05) (Table-I). The clinical efficacy of the ASLC group was significantly higher than that of the SLC group (p≤0.05) (Table-II).

**Table-I:** General characteristics of the two groups.

Characteristics		SLC group (n=53) [ n(%)]	ASLC group (n=53) [ n(%)]	χ^2^/t	p-value
Gender	Male	30 (56.60)	29 (54.72)	0.790^a^	0.433
	Female	23 (43.40)	24 (45.28)		
Age (years)		59.54±6.22	59.12±6.63	0.647^b^	0.511
Lesion diameter (cm)		3.85±0.27	3.83±0.29	0.282^b^	0.770
BMI (kg/m^2^)		22.84±1.36	22.37±1.64	0.478^b^	0.672
Clinical classification	Class III	16 (30.19)	18 (33.96)	0.265^a^	0.631
	Class IV	37 (69.81)	35 (66.04)		

a , indicates statistical value of Chi-square test;

b , indicates statistical value of t test.

**Table-II:** Comparison of clinical effects between the groups (%).

Group	CR [n(%)]	PR [n(%)]	SD [n(%)]	PD [n(%)]	Objective response rate [n(%)]
SLC group (n=53)	5 (14.28)	18 (33.96)	21 (39.62)	9 (16.98)	23 (43.39)
ASLC group (n=53)	11 (20.75)	23 (43.39)	16 (30.19)	3 (5.67)	34 (64.15)
*χ* ^2^	-	-	-	-	11.132
p-value	-	-	-	-	<0.001

**Fig.1:**
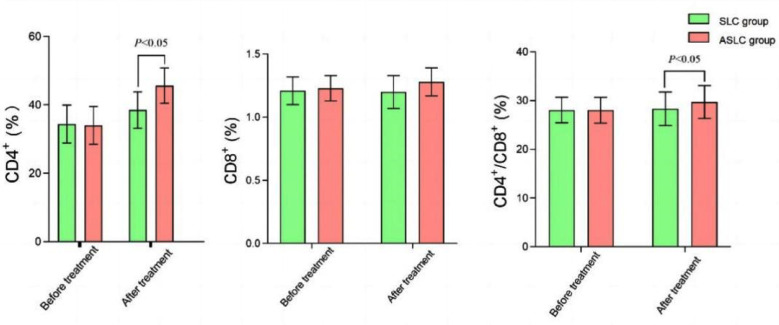
Comparison of immune cell between the groups.

Before treatment, no significant difference was found in the levels of immune cells between the groups (p>0.05). After treatment, patients in the ASLC group exhibited significantly higher levels of CD4+/CD8+ and CD4+ compared to those in the SLC group (p≤0.05), while the difference in CD8^+^ level between the two groups was not statistically significant (p≥0.05) (Fig.1).

**Fig.2:**
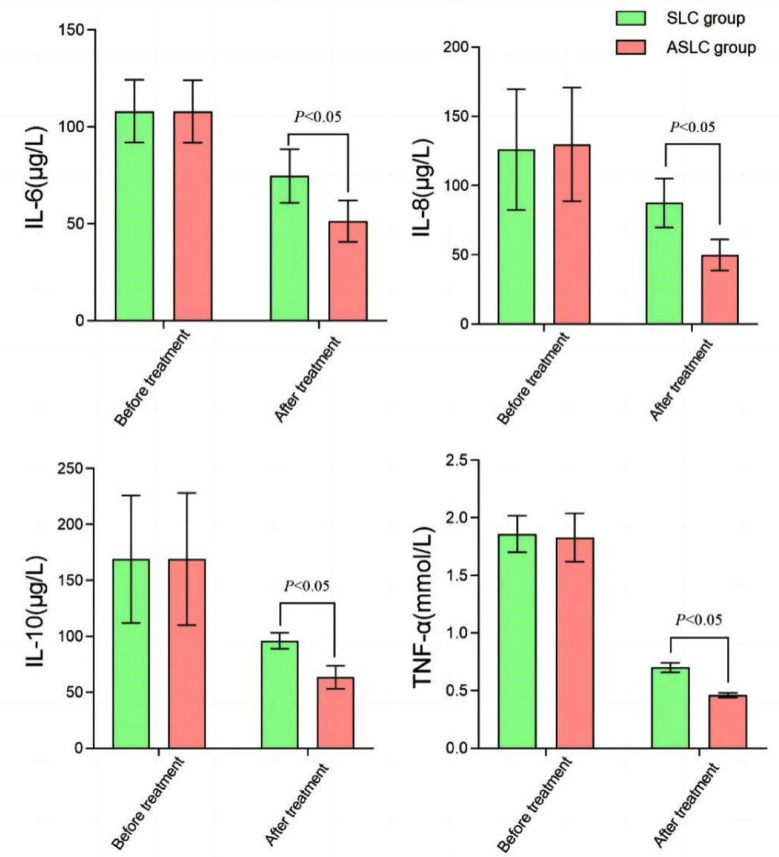
Comparison of inflammatory factors between two groups.

Before treatment, no significant difference was found in the level of immune cells between the two groups (p>0.05). After treatment, levels of TNF-α, IL-10, IL-8, and IL-6 in patients of the ASLC group were lower than those of the SLC group (p≤0.05) Fig.2 No significant difference was found in the incidences of alopecia, liver damage, nausea and vomiting, and leukopenia between the groups (p>0.05) Table-III.

**Table-III:** Comparison of adverse reaction between the groups.

Adverse reactions	SLC group (n=53) [n(%)]	ASLC group (n=53) [n(%)]	χ^2^	p-value
Nausea	9 (16.98)	10 (18.86)	0.543	0.745
Liver damage	3 (5.67)	4 (7.54)	0.000	1.000
Alopecia	7 (13.20)	5 (9.43)	0.752	0.671
Leukopenia	4 (7.54)	3 (5.67)	0.421	0.802
Total rate for adverse reactions	23 (43.39)	22 (41.51)	0.015	0.985

## DISCUSSION

The results of this study showed that the combination of anlotinib and SLC can improve the levels of immune cells and inflammatory factors in patients with advanced NSCLC, and improve their objective response rate. Our results are consistent with previous studies.^13^-^15^ Wang et al, have shown that the combination of anlotinib and chemotherapy may be effective and well-tolerated for advanced NSCLC patients who have failed first-line or SLT.^13^ The study by He et al, also suggests that the combination of anlotinib and pemetrexed as maintenance therapy may be the best choice for treating patients with advanced wild-type EGFR/ALK non-squamous cell carcinoma and NSCLC.^14^ In addition, Li et al, showed good efficacy and tolerable safety of anlotinib combined with anti PD-1 inhibitors in second-line or late-stage treatment of patients with advanced solid tumors.^15^

Numerous studies have showed that the occurrence and development of NSCLC is a multi-factor process, and angiogenesis plays a crucial role is the pathophysiology of invasion and metastasis of this type of cancer.^16^,^17^ Therefore, inhibition of angiogenesis remains the main strategy of LC treatment.^18^ Anlotinib, a multi-target TKI, halts tumor progression by inhibiting cell migration and capillary formation in endothelial cells.^19^ Anlotinib has been demonstrated to be effective in advanced NSCC, and has been approved as a third-line therapy for patients with advanced disease.^20^ However, data of anlotinib as the SLT of advanced NSCC are still scarce.^19^,^20^

Our findings showed a significant superior clinical effect in the ASLC group compared to the SLC group, which is consistent with Wang et al.^13^ In addition, no statistical difference was found in the incidence of adverse reactions between the groups. Moreover, the current study revealed that the adverse reactions of anlotinib combined with SLC were mainly at grade 1-2, with a low incidence of grade III, and no occurrences of grade IV or severe mortality risk. Most symptoms could be relieved or alleviated after reducing the dosage of the drug or by withdrawal, under close monitoring and symptomatic treatment. Our results showed that the combined treatment can improve the clinical effect with safe profile. As shown by recent studies, anti-angiogenic medications can inhibit tumor angiogenesis, promote normalization of tumor blood vessels, enhance the delivery of drugs to tumor tissues, and counteract drug resistance.^21^,^22^

Chemotherapy generally has a damaging effect on the functions of multiple tissues and organs, mainly in the form of immunosuppression, as well as subsequent adverse reactions such as bone marrow suppression and infections.^23^ Studies have also showed that the levels of CD4^+^/CD8^+^ and CD4^+^ in patients with LC decrease after chemotherapy.^24^ However, the present study showed that patients in the ASLC group exhibited significantly higher levels of CD4+/CD8+ and CD4+ compared to those in the SLC group after treatment. Our findings are basically consistent with Chen et al.^25^ Therefore, it is speculated that anlotinib combined with SLC can preserve the immune function of patients with NSCLC to some extent.

Our results also showed that after the treatment, ASLC group showed lower levels of TNF-α, IL-10, IL-8, and IL-6 compared to the SLC group, which is consistent with Zou et al.^26^ IL-6 was shown to act directly on LC cells or facilitate the proliferation of tumor cells indirectly. IL-8 promotes tissue angiogenesis and increases the chances of tumor metastasis.^27^,^28^ At the same time, IL-10 can suppress the proliferation and differentiation of T cells and down-regulate the body’s anti-tumor response.^27^-^29^because of their low selectivity, most small molecule inhibitors of VEGFR2 tyrosine kinase show unexpected adverse effects and limited anticancer efficacy. In the present study, we detailed the pharmacological properties of anlotinib, a highly potent and selective VEGFR2 inhibitor, in preclinical models. Anlotinib occupied the ATP-binding pocket of VEGFR2 tyrosine kinase and showed high selectivity and inhibitory potency (IC50 <1 nmol/L Therefore, it is believed that anlotinib combined with SLC might suppress tumor cell activity. The findings of the current study provided strong evidence for the combination of anlotinib and SLC in patients with NSCLC. Future studies could further compare the combination efficacy of anlotinib with different SLC.

### Limitations

The study is retrospective and the sample size is limited, which may have selection bias. Additionally, the influence of the two regimens on the median survival was not compared, so the efficacy of the treatment warrants validation by more high-quality studies.

## CONCLUSION

Anlotinib combined with standard chemotherapy as a SLT demonstrated effectiveness in regulating the levels of immune cells and inflammatory factors, and has a favorable short-term clinical outcome with manageable adverse reactions in patients with advanced NSCLC.

### Authors’ contributions:

**ZL:** Conceived and designed the study.

**ZL**, **XW**, **CH**, **WL** and **YJ:** Collected the data and performed the analysis.

**ZL:** Was involved in the writing of the manuscript and is responsible for the integrity of the study.

All authors have read and approved the final manuscript.

## References

[1] Bray F, Ferlay J, Soerjomataram I, Siegel RL, Torre LA, Jemal A (2018). Global cancer statistics:GLOBOCAN estimates of incidence and mortality worldwide for 36 cancers in 185 countries. CA Cancer J Clin.

[2] Xue C, Dong H, Chen Y, Lu X, Zheng S, Cui H (2022). Neoadjuvant Immune Checkpoint Inhibitors in Non-Small Cell Lung Cancer. J Coll Physicians Surg Pak.

[3] Jiao J, Li WW, Shang YH, Li XF, Jiao M (2023). Clinical effects of Chemotherapy combined with Immunotherapy in patients with advanced NSCLC and the effect on their nutritional status and immune function. Pak J Med Sci.

[4] Ma G, Chen W, Ma M (2019). Effect of Docetaxel Combined with Cisplatin Preoperative Neoadjuvant Chemotherapy for Stage III NSCLC. J Coll Physicians Surg Pak.

[5] Xian-Jun F, Xiu-Guang Q, Li Z, Hui F, Wan-Ling W, Dong L (2014). ERCC1 and BRCA1 mRNA expression predicts the clinical outcome of non-small cell lung cancer receiving platinum-based chemotherapy. Pak J Med Sci.

[6] Langen DAJ, Johnson ML, Mazieres J, Dingemans AMC, Mountzios G, Pless M (2023). Sotorasib versus docetaxel for previously treated non-small-cell lung cancer with KRASG12C mutation:a randomised, open-label, phase 3 trial. Lancet.

[7] Bottger F, Radonic T, Bahce I, Monkhorst K, Piersma SR, Pham TV Identification of protein biomarkers for prediction of response to platinum-based treatment regimens in patients with non-small cell lung cancer. Mol Oncol.

[8] Liu B, Wang Y, Tian S, Hertzanu Y, Zhao X, Li Y (2020). Salvage treatment of NSCLC recurrence after first-line chemotherapy failure:Iodine-125 seed brachytherapy or microwave ablation?. Thorac Cancer.

[9] Saltos AN, Creelan BC, Tanvetyanon T, Chiappori AA, Antonia SJ, Shafique MR (2023). A phase I/IB trial of binimetinib in combination with erlotinib in NSCLC harboring activating KRAS or EGFR mutations. Lung Cancer.

[10] Health Commission Of The People's Republic Of China N. National guidelines for diagnosis and treatment of lung cancer 2022 in China (English version) (2022). Chin J Cancer Res.

[11] US. Department of Health and Human Services Common Terminology Criteria for Adverse Events (CTCAE) Version 5.

[12] Eisenhauer EA, Therasse P, Bogaerts J, Schwartz LH, Sargent D, Ford R (2009). New response evaluation criteria in solid tumours:revised RECIST guideline (version 1.1). Eur J Cancer.

[13] Wang HY, Chu JF, Zhao Y, Tang H, Wang LL, Zhou MQ (2020). A Trial of the Safety and Efficacy of Chemotherapy Plus Anlotinib vs Chemotherapy Alone as Second- or Third-Line Salvage Treatment for Advanced Non-Small Cell Lung Cancer. Cancer Manag Res.

[14] He Z, Yang X, Ma T, Yang Q, Zhang C, Chen Y (2022). Efficacy and safety of anlotinib combined with carboplatin and pemetrexed as first-line induction therapy followed by anlotinib plus pemetrexed as maintenance therapy in EGFR/ALK wild-type advanced non-squamous non-small cell lung cancer in China:a multicenter, single-arm trial. Transl Lung Cancer Res.

[15] Li SH, Li YW, Li YJ, Liu LB, Zhang Q, Lu D (2023). A Retrospective Study of Anlotinib Combined with Anti-PD-1 Inhibitors in the 2nd or Later-Line Treatment of Advanced Solid Tumors. Int J Gen Med.

[16] Saharinen P, Eklund L, Pulkki K, Bono P, Alitalo K (2011). VEGF and angiopoietin signaling in tumor angiogenesis and metastasis. Trends Mol Med.

[17] Hicklin DJ, Ellis LM (2005). Role of the vascular endothelial growth factor pathway in tumor growth and angiogenesis. J Clin Oncol.

[18] Xu Y, Huang Z, Lu H, Yu X, Li Y, Li W (2019). Apatinib in patients with extensive-stage small-cell lung cancer after second-line or third-line chemotherapy:a phase II, single-arm, multicentre, prospective study. Br J Cancer.

[19] Zhao Y, Wan B, Zhang T, Xu Y, Liu H, Lv T (2019). Irinotecan, topotecan, paclitaxel or docetaxel for second-line treatment of small cell lung cancer:a single-center retrospective study of efficiency comparation and prognosis analysis. Transl Lung Cancer Res.

[20] Liu Y, Li HM, Wang R (2021). Effectiveness and Safety of Adding Bevacizumab to Platinum-Based Chemotherapy as First-Line Treatment for Advanced Non-Small-Cell Lung Cancer:A Meta-Analysis. Front Med (Lausanne).

[21] Si X, Zhang L, Wang H, Zhang X, Wang M, Han B (2019). Management of anlotinib-related adverse events in patients with advanced non-small cell lung cancer:Experiences in ALTER-0303. Thorac Cancer.

[22] Han B, Li K, Wang Q, Zhang L, Shi J, Wang Z (2018). Effect of Anlotinib as a Third-Line or Further Treatment on Overall Survival of Patients With Advanced Non-Small Cell Lung Cancer:The ALTER 0303 Phase 3 Randomized Clinical Trial. JAMA Oncol.

[23] Lin B, Song X, Yang D, Bai D, Yao Y, Lu N (2018). Anlotinib inhibits angiogenesis via suppressing the activation of VEGFR2, PDGFRβand FGFR1. Gene.

[24] Daum S, Hagen H, Naismith E, Wolf D, Pircher A (2020). The Role of Anti-angiogenesis in the Treatment Landscape of Non-Small Cell Lung Cancer - New Combinational Approaches and Strategies of Neovessel Inhibition. Front Cell Dev Biol.

[25] Chen K, Xu Y, Huang Z, Yu X, Hong W, Li H (2023). Sintilimab plus anlotinib as second- or third-line therapy in metastatic non-small cell lung cancer with uncommon epidermal growth factor receptor mutations:A prospective, single-arm, phase II trial. Cancer Med.

[26] Zou B, Jiang H, Liu H (2023). Anlotinib Combined with Anti-PD1 Potentiates Anti-Tumor Immunity via Immunogenic Cell Death and Macrophage Reprogramming. Adv Therap.

[27] Xie C, Wan X, Quan H, Zheng M, Fu L, Li Y (2018). Preclinical characterization of anlotinib, a highly potent and selective vascular endothelial growth factor receptor-2 inhibitor. Cancer Sci.

[28] Abdalla AME, Xiao L, Ullah MW, Yu M, Ouyang C, Yang G (2018). Current Challenges of Cancer Anti-angiogenic Therapy and the Promise of Nanotherapeutics. Theranostics.

[29] Wu D, Nie J, Dai L, Hu W, Zhang J, Chen X (2019). Salvage treatment with anlotinib for advanced non-small cell lung cancer. Thorac Cancer.

